# Supplementary material for: A Likelihood Approach for Real-Time Calibration of Stochastic Compartmental Epidemic Models
Source: PLoS Comput Biol. 2017 Jan 17;13(1):e1005257. doi: 10.1371/journal.pcbi.1005257 (PMC5240920; doi:10.1371/journal.pcbi.1005257)
Supplement: S1 File — (TAR.GZ) [file pcbi.1005257.s014.tar.gz › HSPH_Online-SI-Revision/MSS10/n1-severe/n1-severe_NEW2_summary2.pdf]

peak −8	3w spec	3w cum	5w spec	5w cum	end	3w spec LOG	3w cum LOG	5w spec LOG	5w cum LOG	end LOG
mean	162.9	134.4	113.8	112.9	40.7	0.5	0.4	0.6	0.4	0.3
median	118.2	91.	93.5	89.6	38.2	0.5	0.4	0.5	0.4	0.3
quantile .1	48.6	47.9	41.	43.8	27.3	0.2	0.2	0.2	0.2	0.1
quantile .9	379.6	265.	197.6	183.	55.5	0.8	0.6	1.	0.2	0.7
43	40	39	42	36						

peak −4	3w spec	3w cum	5w spec	5w cum	end	3w spec LOG	3w cum LOG	5w spec LOG	5w cum LOG	end LOG
mean	51.4	44.4	61.3	42.2	40.	0.3	0.2	0.4	0.2	0.3
median	47.2	42.	50.6	40.	35.3	0.2	0.2	0.2	0.2	0.2
quantile .1	25.4	18.7	26.	16.2	22.1	0.1	0.1	0.1	0.1	0.1
quantile .9	79.3	78.	108.6	64.	64.7	0.7	0.3	1.1	0.1	0.7
38	36	32	31	20						

peak	3w spec	3w cum	5w spec	5w cum	end	3w spec LOG	3w cum LOG	5w spec LOG	5w cum LOG	end LOG
mean	60.6	42.	73.4	45.5	51.4	0.3	0.2	0.5	0.2	0.3
median	52.3	36.6	68.4	41.8	51.4	0.3	0.2	0.3	0.2	0.2
quantile .1	24.2	21.6	24.9	23.7	17.2	0.1	0.1	0.1	0.1	0.1
quantile .9	95.1	67.9	121.	58.9	78.8	0.6	0.3	1.2	0.1	0.5
25	22	22	19	15						

peak +4	3w spec	3w cum	5w spec	5w cum	end	3w spec LOG	3w cum LOG	5w spec LOG	5w cum LOG	end LOG
mean	115.	43.1	435.7	47.4	60.5	0.5	0.2	0.8	0.3	0.4
median	56.	39.3	75.6	42.9	55.	0.3	0.2	0.5	0.2	0.3
quantile .1	30.7	17.4	37.5	18.2	28.4	0.1	0.1	0.2	0.1	0.1
quantile .9	78.7	58.5	181.5	62.2	75.5	0.9	0.5	1.6	0.1	0.7
38	34	34	32	26						

peak +8	3w spec	3w cum	5w spec	5w cum	end	3w spec LOG	3w cum LOG	5w spec LOG	5w cum LOG	end LOG
mean	588.2	827.2	329.5	952.7	1033.6	0.7	0.4	0.9	0.5	0.6
median	71.8	48.8	91.4	56.9	65.5	0.5	0.3	0.7	0.4	0.5
quantile .1	43.2	22.	57.9	27.	30.	0.2	0.1	0.1	0.1	0.1
quantile .9	984.	242.4	282.	334.1	416.3	1.2	0.5	1.7	0.1	0.9
31	29	24	23	17	{}	{}	{}	{}	{}	{}
